# Dry Eye Syndrome

**Published:** 2011-07

**Authors:** Mohammad-Ali Javadi, Sepehr Feizi

**Affiliations:** Ophthalmic Research Center, Shahid Beheshti University of Medical Sciences, Tehran, Iran

**Keywords:** Dry Eye Syndromes, Keratoconjunctivitis Sicca

## Abstract

Our understanding of keratoconjunctivitis sicca (KCS), also known as dry eye syndrome, has been changed over recent years. Until lately, the condition was thought to be merely due to aqueous tear insufficiency. Today, it is understood that KCS is a multifactorial disorder due to inflammation of the ocular surface and lacrimal gland, neurotrophic deficiency and meibomian gland dysfunction. This change in paradigm has led to the development of new and more effective medications.

## INTRODUCTION

Dry eye is a disorder of the tear film which occurs due to tear deficiency or excessive tear evaporation; it causes damage to the interpalpebral ocular surface and is associated with a variety of symptoms reflecting ocular discomfort.^1^ Dry eye syndrome, also known as keratoconjunctivitis sicca (KCS), is a common condition reported by patients who seek ophthalmologic care and is characterized by inflammation of the ocular surface and lacrimal glands.

Dry eye symptoms may be a manifestation of a systemic disease, therefore timely detection may lead to recognition of a life–threatening condition. Additionally, patients with dry eye are prone to potentially blinding infections, such as bacterial keratitis^2^ and also at an increased risk of complications following common procedures such as laser refractive surgery.

Knowledge of the pathophysiology of dry eye has recently been improved and the condition is now understood to be a multifactorial disease, characterized by inflammation of the ocular surface and reduction in tear production.^3^ This awareness has led to the development of highly effective therapies.

## EPIDEMIOLOGY

Approximately 1 out of 7 individuals aged 65 to 84 years reports symptoms of dry eye often or all of the time.^4^ Moss et al^5^ reported the prevalence of dry eye to be 14.4% in 3,722 subjects aged 48 to 91 years and noted that the prevalence of the condition doubled after the age of 59. Schein et al^4^, in contrast, found no correlation between dry eye and age or sex, while other researchers have reported such associations to exist. A study on 926 subjects aged 40 years and older, found a higher prevalence of dry eye in women who were also more likely to have a dry eye-related diagnosis or procedure.^6^ According to another study, women experienced a sharp increase in the prevalence of dry eye earlier than men, around the age of 45, roughly at the onset of menopause.^7^

Epidemiological studies on dry eye syndrome suggest vast differences in prevalence. The difficulty in determining the extent of the disease stemmed in part from limited understanding of the pathophysiology of dry eye. As such, definitions of dry eye syndrome differed from one study to another, making results difficult to compare.^8^ This is further complicated by the lack of a standardized clinical testing protocol to diagnose the condition.

## CLINICAL TYPES

The precorneal tear film is an essential component of the ocular surface and can be subdivided into an anterior lipid layer, a middle aqueous layer and an innermost mucin layer. These layers are produced by the meibomian glands, the lacrimal gland and goblet cells of the conjunctiva, respectively.^2^ The tear film lubricates the eye, maintains nutrition and oxygenation of ocular structures, acts as a refractive component and helps remove debris from the ocular surface. In terms of tear production, dry eye can be divided into tear deficient and evaporative types.^3^ Tear deficiency dry eye can further be subdivided into non-Sjogren syndrome and Sjogren syndrome, which is an autoimmune disease associated with lacrimal and salivary gland lymphocytic infiltration. Evaporative dry eye can be divided into meibomian gland disease (MGD) and exposure-related dry eye.^4^,^5^ In yet another group of patients, mucin deficiency due to Stevens-Johnson syndrome or ocular cicatricial pemphigoid is the underlying mechanism of dry eye.^8^

## ETIOLOGY

Dry eye syndrome is associated with a long list of causes which can be divided into primary and secondary. Dry eye may develop secondary to inflammatory disease (e.g. vascular, allergic), environmental conditions (e.g. allergens, cigarette smoke, dry climate), hormonal imbalance (e.g. perimenopausal women and patients under hormone replacement therapy), and contact lens wear. Systemic disorders, such as diabetes mellitus, thyroid disease, rheumatoid arthritis and systemic lupus erythematosus can also lead to dry eye. In addition, neurotrophic deficiency, previous eye surgery (such as corneal transplantation, extracapsular cataract procedures and refractive surgery), or long-term use of medications which create hypersensitivity or toxicity in the eye can predispose to dry eye. Many systemic medications, such as diuretics, antihistamines, antidepressants, psychotropics, cholesterol lowering agents, beta-blockers and oral contraceptives may also be associated with dry eye.^9^,^10^

Postmenopausal women may be the largest at risk group; this is due to a decrease in hormonal levels leading to loss of anti-inflammatory protection and decreased lacrimal secretion.^9^

## PATHOGENESIS

Studies performed on the proteomic profile of the ocular surface comparing dry with normal eyes using enzyme-linked immunosorbent assay (ELISA) has revealed a decrease in lactoferrin and epidermal growth factor in dry eyes. A protein found in acinar cells of the lacrimal gland, AQP-5, was shown to be increased in the Sjogren type of dry eye syndrome, indicating possible leakage of such proteins into the tear film due to lymphocytic infiltration of the lacrimal gland.^9^ Solomon et al^10^ found an increase in inflammatory cytokines interleukin 1 (IL-1) alpha and IL-1 beta in both MGD and Sjogren syndrome, indicating increased protease activity on the ocular surface, mainly in the conjunctival epithelium. Apart from IL-1, IL-6 was also increased in Sjogren syndrome,^11^ indicating an inflammatory process in this subgroup of dry eye. Another study investigating sialic acid, a component of mucin in tears, found a lower level in dry eye patients compared to controls, indicating a change in quantity and quality of tear film glycoproteins in dry eye disease.^12^ The change in tear protein profile in dry eye syndrome, especially in Sjogren disease, has shed light on mechanisms of dry eye.

## CLINICAL SYMPTOMS

Symptoms associated with dry eye may include ocular burning, foreign body sensation, stinging sensation, pain, photophobia and blurred vision.^9^–^12^

## WORKUP

As dry eye syndrome may be associated with a variety of causes, it is important to perform a comprehensive evaluation before proceeding to treatment. A careful history should be obtained with particular attention to diabetes, thyroid disease, connective tissue disorders and contact lens wear. Previous ocular procedures, such as laser refractive surgery are also important in determining the cause of dry eye syndrome. Many medications can affect tear secretion and it is important to review the patient’s drug history. A careful clinical examination should include slit lamp biomicroscopy to determine ocular surface status and diagnose associated meibomian gland dysfunction, blepharitis or meibomian seborrhoea. Examination of the tarsus and fornices for scars and symblepharon is important to exclude pre-existing Stevens-Johnson syndrome, other ocular surface inflammatory disease or previous infections. A careful look at the conjunctiva and cornea would be helpful to assess the severity of dry eye; an increase in staining is observed in more severe cases. Occasionally, corneal filaments and edema may be observed in extremely dry eyes. It is important to be thorough in the examination to find other areas of involvement. Certain systemic causes of dry eye, such as rheumatoid arthritis and systemic lupus erythematosus not only involve the ocular surface but also cause inflammation of the episclera or sclera, and occasionally the posterior segment.

## DIAGNOSTIC CRITERIA

Ohashi et al^9^ suggested that a combination of (1) dry eye symptoms, (2) suggestive findings on Schirmer (< 5 mm wetting after 5 minutes) and fluorescein clearance tests, and (3) fluorescein and Rose Bengal staining (> 3+) would verify clinical dry eye. Other authors have devised different diagnostic criteria and there is no consensus in this regard.^13^–^15^ To further complicate the issue, symptoms and signs do not always correlate well with each other in many patients.^14^

To confirm a diagnosis of dry eye, certain tests are required in the clinical setting. Tear film stability can be assessed with the fluorescein tear break-up time test (TBUT). This measures the interval in seconds between a complete blink and the first appearance of a dry spot or discontinuity in the precorneal film. Patients with TBUT less than 3 seconds are classified with clinical dry eye. If there is aqueous deficiency, the tear meniscus will appear to be thin, less than 1 mm in height. Another clinical method for assessing the severity of dry eye is ocular surface dye staining. Fluorescein and Rose Bengal stains can be used as diagnostic dyes. Fluorescein staining occurs when the epithelial barrier is disrupted and serves as a good test for evaluation of dry eye. Rose Bengal stains devitalized epithelial cells on the conjunctiva and serves a similar purpose. However, Rose Bengal causes transient irritation after instillation and may be less comfortable. Patients with dry eye syndrome can show signs of punctate epitheliopathy and even corneal abrasions.

Another important clinical test is the Schirmer test which measures aqueous tear production. This test is easy to perform in clinical settings but may be subject to errors. Strips of filter paper, called Schirmer strips, are placed on the lower lid inside the tarsal conjunctiva. The patient is allowed to blink normally and the tear strip is scored according to the degree it wets in 5 minutes. There are two ways to perform this test: (a) without topical anesthesia (Schirmer test I) which evaluates the ability of the ocular surface to respond to surface stimulation; and (b) under topical anesthesia (Schirmer test II) which evaluates basal tear secretion. Patients with tear soaking less than 10 mm are considered to have clinical dry eye and eyes with less than 5 mm wetting are diagnosed as severely dry. However, it is important to note that Schirmer tests are subject to environmental and physiologic changes with varying results over time.

## MANAGEMENT

Management of dry eye depends on the cause and severity of the condition. Artificial tears are used to replenish the deficient aqueous layer of the tear film and to dilute inflammatory cytokines. Artificial tears are available in different viscosities and preserved or non-preserved preparations. If the tear deficiency is severe, more viscous agents such as gels or ointments can be used to maintain longer protection. Since KCS, including Sjogren syndrome, is associated with inflammation, the use of topical steroids or non-steroidal anti-inflammatory medications is sometimes helpful. Topical antibiotics may be necessary if the dry eye syndrome is associated with corneal complications. Meibomian gland disease warrants prescription of eyelid hygiene and warm lid compresses, together with topical or even systemic antibiotics such as doxycycline.^13^,^14^

For more severe disease, topical immunomodulating agents, such as cyclosporine-A drops, may become necessary. Studies have demonstrated an improvement in signs and symptoms of dry eye, together with reduction in conjunctival T-cell infiltration and tear cytokine levels following the use of cyclosporine-A drops.^15^,^16^

In very severe cases, frequent topical lubricants may not suffice. Studies have looked into the use of autologous serum as topical eye drops for severely dry eyes with improvement reported after prolonged treatment regimens ranging from 4 to 6 weeks. Growth factors provided by autologous serum are important for epithelial healing. Autologous serum can be prepared by centrifuging venous blood and diluting it with balanced salt solution to 20%.^17^

Bandage contact lenses are sometimes useful in dry eyes to minimize the extent of exposure keratopathy. Severe dry eye disease with corneal complications may warrant surgical intervention such as punctal occlusion. Lacrimal puncta can be plugged temporarily with absorbable collagen plugs or for a longer period of time with non-absorbable plugs which need to be removed if problems arise. Permanent punctal occlusion can also be performed to prevent tears from draining through the drainage system. For patients with dry eye secondary to connective tissue disease, it is important to collaborate with internists and optimize treatment for the systemic disorders. In very severe dry eye secondary to ocular surface disease (such as chemical injury, Stevens-Johnson syndrome, or ocular cicatricial pemphigoid), amniotic membrane transplantation, tarsorrhaphy, keratoplasty, limbal stem cell transplantation, or even ocular prostheses, such as rigid scleral contact lenses, may become necessary for restoration of vision.^18^

### Anti-Inflammatory Therapy

Before recent findings reframed our understanding of dry eye, treatment was limited to the use of artificial tears, ointments, and non-pharmacologic therapies, such as punctal occlusion, environmental control, moisture-retaining eyewear, and surgery. Each had limited efficacy and few resulted in long-lasting improvement in patients’ quality of life. Today, dry eye can be treated successfully with anti-inflammatory agents, the most beneficial of which is topical cyclosporine. Clinical evidence indicates that anti-inflammatory therapy inhibits the production of inflammatory mediators and reduces the signs and symptoms of KCS.

#### Corticosteroids

Corticosteroids are potent anti-inflammatory agents routinely used to control inflammation in various organs. Corticosteroids have multiple mechanisms of action. They work through traditional glucocorticoid receptor mediated pathways which directly regulate gene expression and also by non-receptor pathways that interfere with transcriptional regulators of pro-inflammatory genes. Among their multiple biological activities, corticosteroids inhibit the production of inflammatory cytokines and chemokines, decrease the synthesis of matrix metalloproteinases and lipid mediators of inflammation (e.g. prostaglandins), reduce the expression of cell adhesion molecules (e.g. intercellular adhesion molecule 1), and stimulate lymphocyte apoptosis.^19^–^21^ Steroids have been reported to decrease the production of a number of inflammatory cytokines, including IL-1, IL-6, IL-8, tumor necrosis factor-alpha (TNF-α), and granulocyte macrophage colony-stimulating factor (GM-CSF), and matrix metalloproteinase 9 (MMP-9) by the corneal epithelium.^22^ Corticosteroids have been successfully used for treatment of corneal epithelial disease due to dry eye in several clinical studies.^23^–^25^

#### Cyclosporine

Cyclosporine A (CsA) is a lipophilic cyclic undecapeptide isolated from the fungus *Hypocladium inflatum gams*.^26^ It was first introduced for clinical use as an immune-suppressant drug for prevention of organ transplant rejection in 1983. The immuno modulatory effect of this agent has been proven to be beneficial for treatment of a broad range of disorders such as psoriasis, rheumatoid arthritis and ulcerative colitis which have an underlying inflammatory basis. One of its mechanisms of action is inhibition of calcineurin, a serine/threonine phosphatase, with subsequent reduction in the expression of certain genes involved in T-cell activation such as IL-2, IL-4, IL-12.

The potential of CsA for treating dry eye disease has been demonstrated in several randomized double-masked clinical trials.^27^–^29^

#### Tetracyclines and their Derivatives

The tetracyclines have anti-inflammatory, as well as antibacterial, properties that may make them useful for management of chronic inflammatory diseases. These agents decrease the activity of collagenase, phospholipase A_2_, and several matrix metalloproteinases. They also decrease the production of IL-1α and TNF-α in a wide range of tissues, including the corneal epithelium.^30^–^32^ At high concentrations, tetracyclines inhibit staphylococcal exotoxin-induced cytokines and chemokines.^33^,^34^ Tetracyclines are also known to inhibit matrix metalloproteinase expression, suggesting a rationale for their use in ocular rosacea.^35^ They can also inhibit angiogenesis, which may develop in rosacea.

Doxycycline was discovered in the early 1960s. It is a semisynthetic long-acting tetracycline derivative which inhibits bacterial ribosomes in a wide variety of micro-organisms. In subantimicrobial doses it is also effective as primary treatment for rosacea and sterile corneal ulceration.^36^,^37^ Previous studies using experimental dry eye models demonstrated that doxycycline was efficacious in decreasing gelatinolytic activity in the ocular surface epithelia, as well as decreasing levels of MMP-9 mRNA transcripts, and preventing experimental dry eye-induced increase in IL-1 and TNF-α.^38^ Doxycycline also improves corneal surface regularity and barrier function.^39^

#### Essential Fatty Acids

Essential fatty acids are necessary for health. They cannot be synthesized by vertebrates and must be obtained from dietary sources. Among the essential fatty acids are the 18 carbon omega-6 and omega-3 fatty acids. In a typical western diet, omega-6 fatty acids are consumed 20 to 25 times more than omega-3 fatty acids. Omega-6 fatty acids are precursors of arachidonic acid and certain pro-inflammatory lipid mediators such as prostaglandin E_2_ and leukotriene B_4_. In contrast, certain omega-3 fatty acids, such as eicosapentaenoic acid found in fish oil, inhibit synthesis of these lipid mediators and also block the production of IL-1 and TNF-α.^40^,^41^ A beneficial clinical effect from fish oil omega-3 fatty acids on rheumatoid arthritis has been observed in several double-masked placebo-controlled clinical trials.^42^,^43^ In a prospective placebo-controlled clinical trial, linoleic acid and gamma-linoleic acid administered orally twice a day led to significant improvement in ocular irritation symptoms, and a reduction in ocular surface lissamine green staining conjunctival HLA-DR reactivity.^44^ In an animal model of induced dry eye, topical treatment with alpha-linolenic acid significantly decreased corneal fluorescein staining as compared to both vehicle and untreated controls, decreased CD11b(+) cells and reduced the expression of corneal IL-1α and TNF-α, and conjunctival TNF-α.^45^

## CONCLUSIONS

Dry eye syndrome consists of a wide spectrum of disorders with different causes. Clinicians should be aware of the extent of dry eye symptoms. A thorough history and investigation is necessary to identify the cause of dry eye. Useful clinical tests for assessing the severity of the condition include Schirmer, fluorescein dye, and tear break up time tests. Management depends on an accurate diagnosis and the severity of the condition. Treatments that replenish deficient tears include artificial tears, gels and ointments in mild to moderate disease. Other treatment modalities such as topical steroids, immunomodulating drugs, antibiotics, bandage contact lenses, autologous serum and amniotic membrane transplantation may be used in more severe cases. In severely dry eyes, surgical intervention such as punctal occlusion can be employed to minimize tear drainage. Certain conjunctival and lid operations can also be performed to treat specific causes.
